# Supplementary material for: MetaRibo-Seq measures translation in microbiomes
Source: Nat Commun. 2020 Jun 29;11:3268. doi: 10.1038/s41467-020-17081-z (PMC7324362; doi:10.1038/s41467-020-17081-z)
Supplement: Supplementary file 10 — Supplementary Data 7 [file 41467_2020_17081_MOESM10_ESM.zip › File2/Confidence_VeryHigh_Taxonomy/3213_out.krona.html]

Javascript must be enabled to view this page.

members
magnitude
magnitudeUnassigned
count
unassigned
taxon
rank

3213\_out

18

2
superkingdom
18

1239
18
phylum

186801
18
class

18
order
186802

186806
3
family

1730
genus
3

1262884

SRS144297\_contig\_number\_13697SRS144714\_contig\_number\_contig-100\_306.134683SRS893383\_contig\_number\_contig-100\_4778.4779
species
3

541000
15
family


SRS013940\_contig\_number\_16855SRS015782\_contig\_number\_41834SRS043768\_contig\_number\_28714SRS047044\_contig\_number\_14419SRS077231\_contig\_number\_contig-100\_823.138310SRS097920\_contig\_number\_30464SRS1041090\_contig\_number\_4731SRS104197\_contig\_number\_contig-100\_28.28SRS1055022\_contig\_number\_19204SRS143148\_contig\_number\_contig-100\_283.124702SRS147766\_contig\_number\_11008SRS148784\_contig\_number\_14568SRS893366\_contig\_number\_13054SRS893369\_contig\_number\_7063
1898205
14
species

1263
1
genus

species
1

SRS014313\_contig\_number\_5628
2293177
